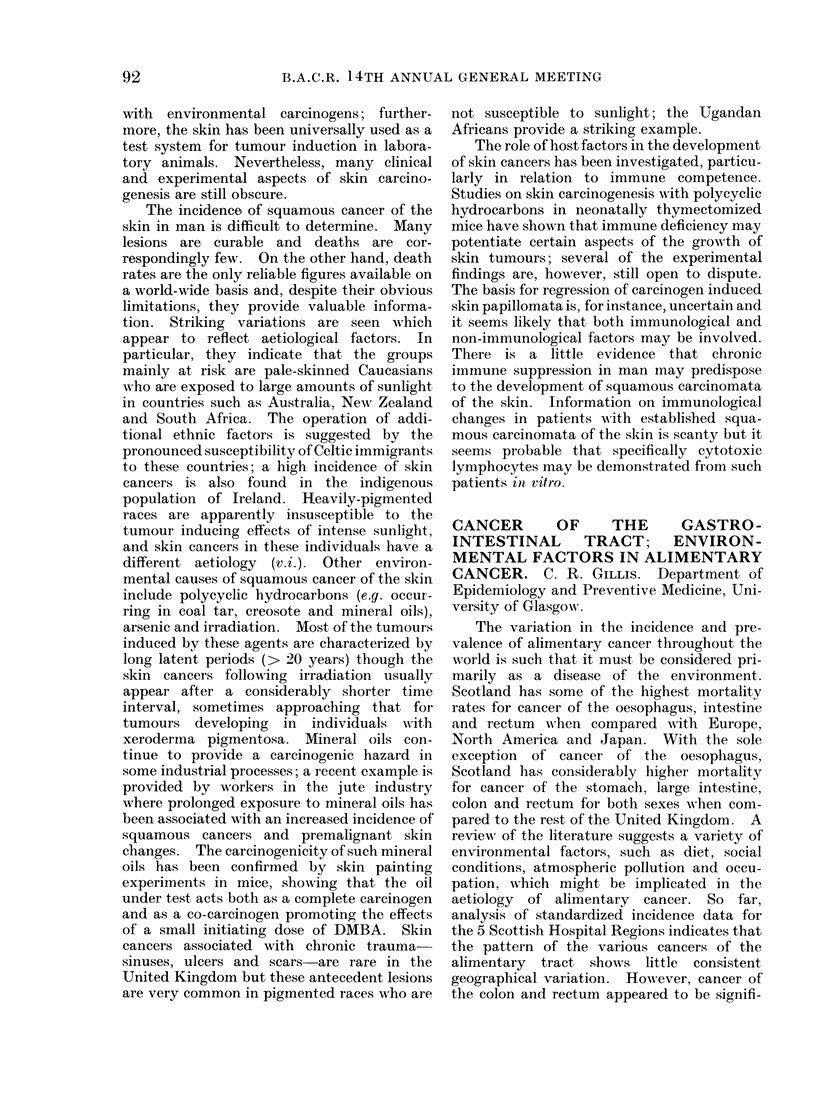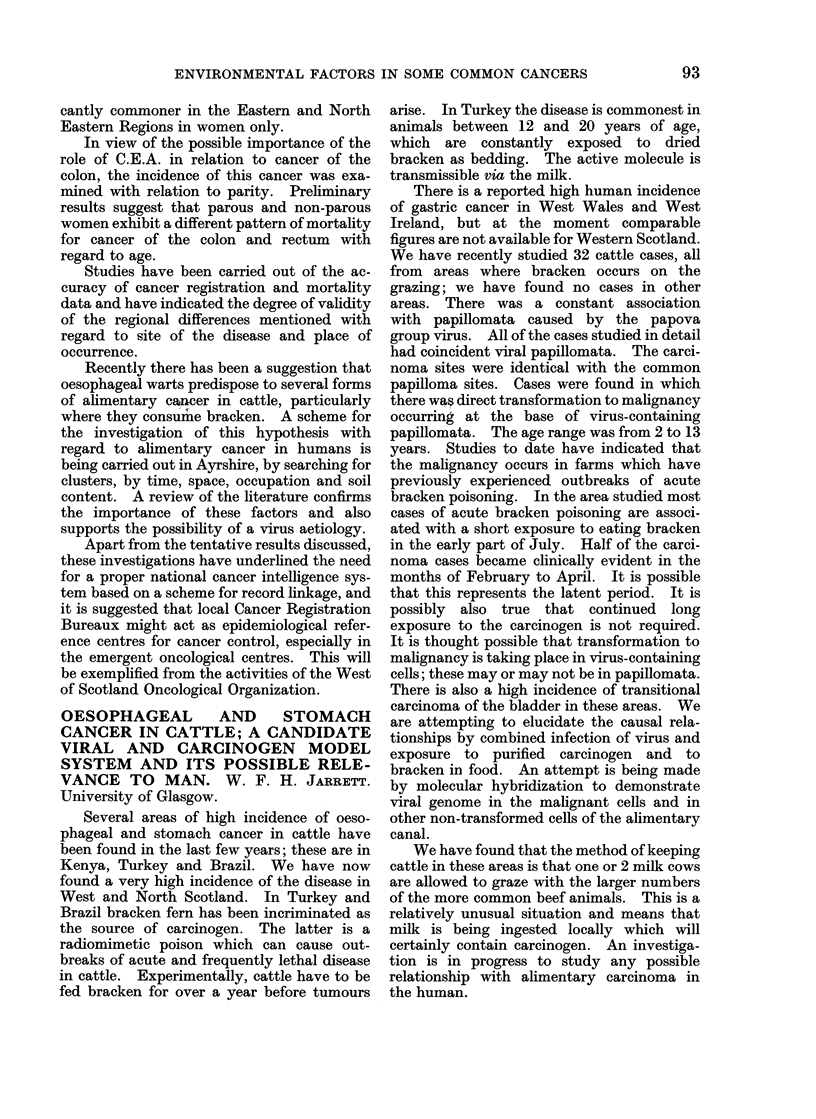# Cancer of the gastrointestinal tract; environmental factors in alimentary cancer.

**DOI:** 10.1038/bjc.1973.120

**Published:** 1973-07

**Authors:** C. R. Gillis


**CANCER OF THE GASTRO-INTESTINAL TRACT; ENVIRONMENTAL FACTORS IN ALIMENTARY CANCER.** C. R. GILLIS. Department of Epidemiology and Preventive Medicine, University of Glasgow.

The variation in the incidence and prevalence of alimentary cancer throughout the world is such that it must be considered primarily as a disease of the environment. Scotland has some of the highest mortality rates for cancer of the oesophagus, intestine and rectum when compared with Europe, North America and Japan. With the sole exception of cancer of the oesophagus, Scotland has considerably higher mortality for cancer of the stomach, large intestine, colon and rectum for both sexes when compared to the rest of the United Kingdom. A review of the literature suggests a variety of environmental factors, such as diet, social conditions, atmospheric pollution and occupation, which might be implicated in the aetiology of alimentary cancer. So far, analysis of standardized incidence data for the 5 Scottish Hospital Regions indicates that the pattern of the various cancers of the alimentary tract shows little consistent geographical variation. However, cancer of the colon and rectum appeared to be significantly commoner in the Eastern and North Eastern Regions in women only.

In view of the possible importance of the role of C.E.A. in relation to cancer of the colon, the incidence of this cancer was examined with relation to parity. Preliminary results suggest that parous and non-parous women exhibit a different pattern of mortality for cancer of the colon and rectum with regard to age.

Studies have been carried out of the accuracy of cancer registration and mortality data and have indicated the degree of validity of the regional differences mentioned with regard to site of the disease and place of occurrence.

Recently there has been a suggestion that oesophageal warts predispose to several forms of alimentary cancer in cattle, particularly where they consume bracken. A scheme for the investigation of this hypothesis with regard to alimentary cancer in humans is being carried out in Ayrshire, by searching for clusters, by time, space, occupation and soil content. A review of the literature confirms the importance of these factors and also supports the possibility of a virus aetiology.

Apart from the tentative results discussed, these investigations have underlined the need for a proper national cancer intelligence system based on a scheme for record linkage, and it is suggested that local Cancer Registration Bureaux might act as epidemiological reference centres for cancer control, especially in the emergent oncological centres. This will be exemplified from the activities of the West of Scotland Oncological Organization.